# *Anaplasma phagocytophilum *in ticks in Slovenia

**DOI:** 10.1186/1756-3305-3-102

**Published:** 2010-11-04

**Authors:** Katja Strašek Smrdel, Mojca Serdt, Darja Duh, Nataša Knap, Tatjana Avšič Županc

**Affiliations:** 1 Institute of Microbiology and Immunology, Faculty of Medicine, Zaloška 4, SI-1000 Ljubljana, Slovenia; 2 Public Health Institute Maribor, Prvomajska 1, 2000 Maribor, Slovenia

## Abstract

Ticks act as vectors of many pathogens of domestic animals and humans. *Anaplasma phagocytophilum *in Europe is transmitted by the ixodid tick vector *Ixodes ricinus*. *A. phagocytophilum *causes a disease with diverse clinical signs in various hosts. A great genetic diversity of the *groESL *operon of *A. phagocytophilum *has been found in ticks elsewhere. In Slovenia, the variety of the *groESL *operon was conducted only on deer samples. In this study, the prevalence of infected ticks was estimated and the diversity of *A. phagocytophilum *was evaluated. On 8 locations in Slovenia, 1924 and 5049 (6973) *I. ricinus *ticks were collected from vegetation in the years 2005 and 2006, respectively. All three feeding stages of the tick's life cycle were examined. The prevalence of ticks infected with *A. phagocytophilum *in the year 2005 and in the year 2006 was 0.31% and 0.63%, respectively, and it did not differ considerably between locations. The similarity among the sequences of *groESL *ranged from 95.6% to 99.8%. They clustered in two genetic lineages along with *A. phagocytophilum *from Slovenian deer. One sequence formed a separate cluster. According to our study, the prevalence of *A. phagocytophilum *in ticks is comparable to the findings in other studies in Europe, and it does not vary considerably between locations and tick stages. According to *groESL *operon analysis, two genetic lineages have been confirmed and one proposed. Further studies on other genes would be useful to obtain more information on genetic diversity of *A. phagocytophilum *in ticks in Slovenia.

## Findings

Ticks and tick-borne diseases affect animal and human health worldwide. A vector of many diseases in Europe and Slovenia is *Ixodes ricinus *[1]. It can be found in the forest, in shrubby or wooded pastures and on surfaces with low vegetation [2]. Ticks' feeding cycle includes three stages: larva, nymph and adult. *I. ricinus *feeds on livestock, deer, dogs and a wide variety of other species, including humans [2]. *I. ricinus *is a confirmed vector of the bacterium *Anaplasma phagocytophilum *[3]. The tick becomes infected as it feeds on an infected host. Anaplasmae are transmitted from stage to stage as the tick moults (trans-stadially), but not transovarially. No anaplasmae have been detected in unfed larvae so far [4]. *A. phagocytophilum*, the agent of granulocytic anaplasmosis, was formerly known as human granulocytic ehrlichiosis agent (HGE agent), *Ehrlichia phagocytophila *and *E. equi *[5]. *A. phagocytophilum *causes a disease with diverse clinical signs in various hosts from asymptomatic to life-threatening [4]. No fatal infection in humans has been documented in Europe so far. On the contrary, in the USA, the fatality rate in humans is 1% [4]. Important reservoir hosts of the bacterium are small mammals and deer. Humans, dogs, horses represent accidental hosts [4]. The wild boar is suggested as a reservoir host for a variant that infects humans [6]. The prevalence of infected ticks in Europe ranges from 0.4% - 66.7% [7]. To describe the diversity of *A. phagocytophilum*, the *groESL *operon is widely used as the *16 S rRNA *gene is too conservative [8]. It has been shown that, based on this operon, anaplasmae among deer in Slovenia cluster in two genetic lineages [9]. An immense diversity of *groESL *sequences of *A. phagocytophilum *in ticks has also been described in Germany [10]. The variants matched to the sequences found in a German and a Swedish horse and in a Slovenian patient [10]. In a previous study in Slovenia, the estimated prevalence of infected ticks from one location was 3.2% [11]. The ticks in the present study were being collected every month for two years from several locations. The prevalence of ticks infected with *A. phagocytophilum *was estimated and the diversity of the *groESL *operon of detected DNA of *A. phagocytophilum *was evaluated.

The study was performed in the years 2005 and 2006 at 8 locations in Slovenia. The criteria for selecting the locations were the tick-borne pathogens' presence in human patients or a higher altitude of the location compared to others. Ticks were collected at forest edges by dragging a flag with a surface of 1 m^2 ^over 100 m of vegetation [1]. Every 2.5 m, the flag was examined for ticks. The species, stage and sex of ticks were determined by a professional entomologist. Ticks were decontaminated in 70% ethanol and sterile double distilled water and pooled in groups of 30 larvae, 10 nymphs or 5 adults. Adult ticks were first cut in half and a half of each adult tick was used for pooling. The remaining half of the dissected adult tick was frozen and stored separately. Pools of ticks were stored at -20°C until further analysis. The pooled samples were used for DNA extraction. First, they were homogenized using TissueLyser (Retsch for Qiagen, Hilden, Germany). DNA was extracted with BioSprint 15 DNA Blood Kit according to the manufacturer's instructions (Qiagen, Hilden, Germany). To assess the efficiency of DNA extraction, tick mitochondrial *16 S rRNA *was examined [12]. For the initial screening of samples, primer pair Ehr521 and Ehr790, specific for the *16 S rRNA *of genus *Anaplasma *sp. and *Ehrlichia *sp., was used [11]. All positive samples were additionally tested for the *groESL *operon. A nested PCR would amplify a 1296-bp fragment of *groESL *operon of *A. phagocytophilum *variants [8]. *A. phagocytophilum*, grown in a HL-60 cell culture, was used as a PCR positive control. If a pool of adult ticks was positive, the stored half of each dissected tick from a pool was used for DNA extraction and further amplifications. All amplicons of *groESL *operon were further analyzed by sequencing on both strands with the BigDye Terminator Cycle Sequencing Ready Reaction Kit (Applied Biosystems, Foster City, CA, USA). The sequences were analyzed with computer programs of the Lasergene 1999 software package (DNASTAR, Madison, WI, USA) based on Clustal W algorithm [13]. The distance matrices were calculated using Kimura two-parameter method 1980 [14] and the Neighbor-joining method [15] was used for the construction of a phylogenetic tree with TreeCon software (Yves Van de Peer, Department of Biochemistry, University of Antwerp, Antwerpen, Belgium). The stability of inferred topology was assessed with 1000 bootstrap replicates. The prevalence of infection was calculated using the program PooledInfRate version 3.0 (a Microsoft^® ^Excel Add-In, developed by Brad Biggerstaff, CDC, Fort Collins, CO). Statistical analyses were performed using SPSS version 17.0 (SPSS Inc., Chicago, IL). P values of 0.05 or less were considered statistically significant.

On 8 locations in Slovenia, 1924 and 5049 (6973) *I. ricinus *ticks were collected by flagging vegetation in the years 2005 and 2006, respectively. Ticks were separated into pools: 252 pools in 2005 and 442 pools in 2006 (Table 1). At the location of Murska Šuma, other tick species were collected, namely *Dermacentor reticulatus *and *Haemaphysalis concinna*. As *A. phagocytophilum *is transmitted by *Ixodes *spp. ticks [16], only *I. ricinus *was examined.

**Table 1:** *A. phagocytophilum *in pools of ticks and its prevalence in Slovenian I. ricinus

				Adult		
					
Year	Location	Larvae	Nymphs	Male	Female	Total
**2005**	Črni Kal	3	13	2	2	20
	Sodražica	1	14/1 (0.80)	5	4	24/1 (0.63)
	Murska šuma	0	2	4	7	13
	Rakovnik	2	15	12	10	39
	Mozirje	3	23/3 (1.45)	10	8/1 (3.5)	44/4 (1.24)
	Kamniška Bistrica	9	22	9	7	47
	Štefanja Gora	3	27	9/1 (2.97)	6	45/1 (0.28)
	Osolnik	1	10	5	4	20
	**Total**	22	126/4 (0.34)	56/1 (0.53)	48/1 (0.69)	252/6 (**0.31**)
**2006**	Črni Kal	NT	43/5 (1.31)	4	2	49/5 (1.24)
	Sodražica	NT	24/1 (0.43)	8	6	38/1 (0.34)
	Murska šuma	NT	1	10	22	33
	Rakovnik	NT	32/2 (0.65)	17	14	63/2 (0.44)
	Mozirje	NT	41/3 (0.79)	14/2 (3.66)	13/1 (1.97)	68/6 (1.24)
	Kamniška Bistrica	NT	34/2 (0.60)	17	18	69/2 (0.41)
	Štefanja Gora	NT	50/1 (0.21)	13	12	75/1 (0.17)
	Osolnik	NT	25/3 (1.28)	12	10	47/3 (0.90)
	**Total**	NT	250/17 (0.73)	95/2 (0.47)	97/1 (0.23)	442/20 (**0.63**)

In parenthesis in %. NT - not tested. Ticks were sorted into pools of 10 nymphs, 30 larvae or pools of 5 halves of male or female adult ticks.

The *16 S rRNA *of *Anaplasma *sp. was detected in 26 pools of adult and nymphal stages of ticks (Table 1). None of the pool of larvae were positive in the year 2005, and were not tested in the year 2006 as *A. phagocytophilum *is not transovarially transmitted in ticks (Table 1). One adult tick from each pool was positive. The prevalence of infection in the year 2005 and in the year 2006 was 0.31% and 0.63%, respectively (Table 1). No statistically significant differences were found between the prevalences at various locations and in both years (p > 0.05).

**Figure 1:**
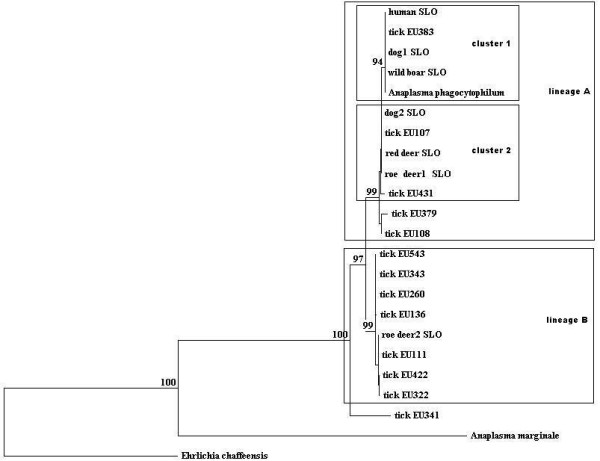
**Phylogenetic relationship of anaplasmae deposited in GenBank and detected in this study in tick samples**. GenBank accession numbers: Slovenian patient [GenBank:AF033101], dog1 [GenBank:EU381150], dog2 [GenBank:EU381151], wild boar [GenBank:EU184703], red deer [GenBank:AF478562], roe deer1 [GenBank:AF478558], roe deer2 [GenBank:AF478564], *A. phagocytophilum *[GenBank:AY529490], *A. marginale *[GenBank:AF414865], *Ehrlichia chaffeensis *[GenBank:L10917]. The number on each branch shows the percent occurrence in 1,000 bootstrap replicates. Only different *groESL *sequences from *I. ricinus *are shown.

The sequences of 26 PCR amplicons of the *groESL *operon matched *A. phagocytophilum*. The similarity varied from 95.6% to 99.8%. Twenty-five sequences of the *groESL *operon clustered in two genetic lineages, A and B (Figure 1). In the genetic lineage A, most of the sequences gathered in two clusters, 1 and 2. Ten sequences of the *groESL *operon were 100% identical to each other and grouped together with a reference sequence from a Slovenian dog [GenBank:EU381151] in cluster 2. Three 100% identical sequences clustered together with a sequence from a Slovenian patient [GenBank:AF033101] in cluster 1 of the same lineage. In the lineage B, three sequences were 100% identical to each other and were identical to a reference sequence from a German tick [GenBank:AY281794] (Table 2). One sequence (tick EU341) from a pool of nymphs showed 95.6% similarity to other sequences from Slovenian ticks and 96% similarity with a reference sequence [GenBank:AY281848] from a German tick and did not cluster in any of the lineages A or B. Only the sequences that were not 100% identical were included in the phylogenetic study.

**Table 2:** Similarity of *groESL *operon sequences between *A. phagocytophilum *in Slovenian ticks and GenBank reference sequences

GenBank accession number	Source	Similarity	Location	Ticks developmental stage	Slovenian sequences from ticks
EU381151	dog, Slovenia	100%	Mozirje	Nymphs	EU107
				Female	EU112, EU412
				Male	EU418
			Štefanja Gora	Nymphs	EU367
				Male	EU132
			Črni kal	Nymphs	EU254, EU277
			Kamniška Bistrica	Nymphs	EU433
			Rakovnik	Nymphs	EU652
AY281794	tick, Germany	100%	Mozirje	Nymphs	EU111
			Sodražica	Nymphs	EU329
			Črni kal	Nymphs	EU622
AY281844	tick, Germany	100%	Mozirje	Nymphs	EU108
		99%	Osolnik	Nymphs	EU379
		99%	Sodražica	Nymphs	EU136
AF478560	roe deer, Slovenia	100%	Črni kal	Nymphs	EU260
AY281848	tick, Germany	96%	Mozirje	Nymphs	EU341
AY281851	tick, Germany	99%	Mozirje	Nymphs	EU343
AF033101	human, Slovenia	100%	Osolnik	Nymphs	EU383, EU381
			Rakovnik	Nymphs	EU295
AY281771	tick, Germany	100%	Mozirje	Nymphs	EU422
AY220470	tick, Austria	100%	Črni kal	Nymphs	EU322
AF548386	sheep, Norway	100%	Kamniška Bistrica	Nymphs	EU431
AY281819	Tick, Germany	99%	Mozirje	Nymphs	EU543

Ticks are the vectors of many disease-causing pathogens. *I. ricinus*, the vector of *A. phagocytophilum*, is distributed throughout Europe, including Slovenia [1]. Environmental factors, such as climate, vegetation type, and abundance of appropriate hosts, influence the geographical distribution of pathogen vectors and, consequently, pathogens themselves [17]. In this study, ticks were collected at 8 different locations in Slovenia in two consecutive years. The overall prevalence of *A. phagocytophilum *infection in ticks in 2005 and 2006 was 0.31% and 0.63%, respectively, which is in agreement with the results from elsewhere in Europe [7]. In a previous study in Slovenia, the estimated prevalence of infected ticks was 3.2%, but this was most likely due to the fact that only a small number of adult ticks was collected at a single location in central Slovenia [11]. There are also some reports of higher prevalence of infected ticks, but the likely reasons are the geographical differences and the presence of appropriate hosts [7], as well as different screening methods used. The infection rate in Slovenia did not differ considerably between the tick stages (Table 1). In the year 2005, no larvae were found positive, which is in concordance with the fact that anaplasmae are not transovarially transmitted [4].

Only a few reports describe the diversity of *A. phagocytophilum *in infected ticks sampled from vegetation [10]. The variety of *groESL *operon sequences has been determined and some matched human and horse cases of anaplasmosis [10]. In this study, a high diversity of sequences of the *groESL *operon in ticks has been found. As discussed in a previous study of deer sequences of *A. phagocytophilum *[9], the sequences from the ticks in Slovenia are also delineated in two genetic lineages (Figure 1). The similarity between these sequences varied from 97.8% to 99.8%. In the lineage A (dog, human, wild boar, tick, deer samples), the similarity ranged from 99.5% to 99.9%, and in the lineage B (tick and deer samples), it varied from 99.0% to 99.8%. Different genetic lineages represent also differences in the amino acid sequence of GroESL protein (an amino acid serine (lineage A) is substituting alanine (lineage B) at the position 242). It is suggested that the strains of *A. phagocytophilum *that possess a variant of the protein with the serine might be pathogenic to humans [18].

A greater diversity of the *groESL *operon was found at the nymphal stage of ticks. For this, many reasons are possible. The *groESL *genetic variants other than those that cause the disease in humans and dogs in Slovenia might not be pathogenic to aforementioned hosts since they have not been found in them yet [11,19]. It is nevertheless possible that they circulate only among small mammals and deer. However, the main reason could be the number of collected ticks: more nymphs than adult ticks were collected at all locations and, consequently, higher diversity was found.

In one pool of nymphs from Mozirje, a *groESL *sequence showed only a 95.6% similarity with other *groESL *operon sequences from the ticks in Slovenia. It did not cluster within the lineages A and B. Moreover, after the translation into amino acid sequence, no difference from the lineage B was found (alanine at the position 242). Probably, a novel genetic lineage of the *groESL *operon of *A. phagocytophilum *was found. To obtain more information about genetic diversity of *A. phagocytophilum *in *I. ricinus *in Slovenia, additional genetic markers, such as *ankA *and *msp4*, should be analyzed.

*I. ricinus *nymphal and adult stages are responsible for the transmission of the pathogen as *A. phagocytophilum *was present in both stages. There was no significant difference in the prevalences of *A. phagocytophilum *at different locations and in both years. The prevalence of infection in ticks did not differ considerably from the reports from elsewhere in Europe. With this study, we have confirmed that *I. ricinus *is a vector of a variant of *A. phagocytophilum *that causes the disease in humans and dogs. The sequencing of the *groESL *operon has demonstrated a great diversity of *A. phagocytophilum *in Slovenia. With phylogenetic analysis, two genetic lineages have been confirmed and another has been proposed. Further phylogenetic studies of several other genes, such as *ankA *and *msp4*, might be useful to obtain more information about genetic diversity.

## Competing interests

The authors declare that they have no competing interests.

## Authors' contributions

KSS conducted the laboratory study and drafted the manuscript. MS was involved in laboratory study. DD facilitated the molecular laboratory study. NK conducted the field study and did the statistical analysis. TAZ was involved in the project design and participated in drafting the manuscript. All co-authors have read the manuscript.
